# Supplementary material for: Zero modes activation to reconcile floppiness, rigidity, and multistability into an all-in-one class of reprogrammable metamaterials
Source: Nat Commun. 2024 Apr 10;15:3087. doi: 10.1038/s41467-024-47180-0 (PMC11006655; doi:10.1038/s41467-024-47180-0)
Supplement: Supplementary file 1 — Supplementary Information [file 41467_2024_47180_MOESM1_ESM.pdf]

Supplementary Information for

Zero modes activation to reconcile floppiness, rigidity, and multistability into an all-in-one class of reprogrammable metamaterials

Lei Wu and Damiano Pasini*

Department of Mechanical Engineering, McGill University, Montreal, Quebec H3A 0C3, Canada

*Corresponding author: Damiano Pasini; damiano.pasini@mcgill.ca

Contents

Supplementary Note 1: Theoretical model for investigating the activation of the metahinge **3**

Supplementary Note 2: Characterization of zero modes of the lattice analogy **8**

Supplementary Note 3: Finite element simulation of biaxial zero-energy modes **10**

Supplementary Note 4: Minimum energy path of the periodic Kagome lattice with selectively added NNN bonds **13**

Supplementary Note 5: Phonon spectrum of the periodic Kagome lattice with selectively added NNN bonds **20**

Supplementary Note 6: Phonon spectrum evolution of the lattice exhibiting non-isochoric reconfiguration **23**

Supplementary Note 7: Geometry of experimental specimens **25**

Supplementary Note 8: Supplementary Movies **27**

Supplementary Note 1: Theoretical model investigating the metahinge activation

The activation process of the metahinge architecture shown in Fig.1b exhibits reflection symmetry with respect to its two central axes. As a result, we extract its top-left quarter and apply sliding boundary conditions to the vertices lying on these two symmetry axes (x and y) shown in Supplementary Figure 1. The coordinates of each vertex are defined in the Cartesian coordinate system $x-o-y$. To describe the kinematics of the system, we assume that the sub-parts ADEFG and ABC can move independently; we use A^+ to denote the A vertex on the sub-part ADEFG and A^- for the sub-part ABC.

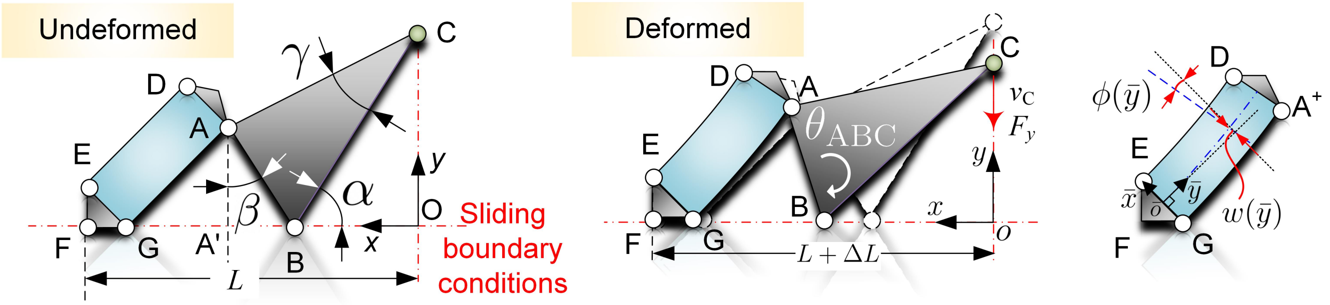

Supplementary Figure 1. Kinematics of the transformable architecture for metahinge activation. From left to right are the top-left quarter of the transformable architecture in its undeformed configuration, deformed configuration, and the deformation of the Timoshenko beam; $\overline{\mathrm{FG}} = \overline{\mathrm{EF}} = 2.75$ mm, $\overline{\mathrm{A'G}} = \overline{\mathrm{AA'}} = 6.50$ mm, $\overline{\mathrm{EG}} = \overline{\mathrm{AD}} = \sqrt{2} * \overline{\mathrm{EF}}$, $\overline{\mathrm{DE}} = \overline{\mathrm{AG}} = \sqrt{2} * \overline{\mathrm{AA'}}$, $L = 22.00$ mm, $\overline{\mathrm{A'B}}$ and $\overline{\mathrm{OC}}$ are design variables; the green spot marked in Fig. 1e corresponds to $\overline{\mathrm{A'B}} = 4.55$ mm and $\overline{\mathrm{OC}} = 14.00$ mm; the Young's modulus, Poisson's ratio, and out-of-plane thickness of the Timoshenko beam ADEG are set to be 146 MPa, 0.47, and 30 mm, respectively [1].

Under the activation force F_y, the triangle EFG undergoes only rigid-body translation along the $x-$axis (Supplementary Figure 1), which is denoted as ΔL. The elastic deformation of the rectangle ADEG is described using the Timoshenko beam model; its edge EG is tied on the hypotenuse of the isosceles right triangle EFG; its end edge $\mathrm{A}^+\mathrm{D}$ remains flat during deformation (right of Supplementary Figure 1). The deflection of the neutral axis $w(\bar{y})$ of the Timoshenko beam $\mathrm{A}^+\mathrm{DEG}$ and the rotation of the cross-section $\phi(\bar{y})$ are defined in a local coordinate system

$\bar{x}-\bar{o}-\bar{y}$. $w(\bar{y})$ and $\phi(\bar{y})$ can be expressed as a superposition of a series of admissible functions:

$$w(\bar{y}) = \sum_{m=1}^{M} A_m * \bar{y}^m * \overline{\mathrm{AG}}^{-m}, \tag{S1}$$

$$\phi(\bar{y}) = \sum_{n=1}^{N} B_n * \bar{y}^n * \overline{\mathrm{AG}}^{-n}, \tag{S2}$$

where M and N are the number of modes taken into account, A_m and B_n are the amplitude of each mode, and $\overline{\mathrm{AG}}$ is the distance between vertices A and G in the undeformed configuration; the overbar convention will now be used in the following to denote the distance between two vertices in the undeformed configuration. In the local coordinate system $\bar{x}-\bar{o}-\bar{y}$, the displacement of the vertex A^+ along the $\bar{y}$-axis is $w_{\bar{y}=\overline{\mathrm{AG}}}$; the projection to the $\bar{x}$-axis is $\phi_{\bar{y}=\overline{\mathrm{AG}}} * \overline{\mathrm{AD}}/2$.

The absolute displacement of vertex A^+ in the global coordinate system $x-o-y$ comprises the relative motion in the local coordinate system $\bar{x}-\bar{o}-\bar{y}$ and the translation associated with triangle EFG:

$$\mathbf{u}_{\mathrm{A}^+} = \mathbf{R} \cdot [w_{\bar{y}=\overline{\mathrm{AG}}}, \phi_{\bar{y}=\overline{\mathrm{AG}}} * \overline{\mathrm{AD}}/2]^{\mathrm{T}} + [\Delta L, 0]^{\mathrm{T}}, \tag{S3}$$

where the rotation matrix $\mathbf{R}$ is given by

$$\mathbf{R} = \begin{bmatrix} \sqrt{2}/2, & \sqrt{2}/2 \\ \sqrt{2}/2, & -\sqrt{2}/2 \end{bmatrix}. \tag{S4}$$

Therefore, the position vector of vertex A^+ in the deformed configuration is

$$\mathbf{x}_{\mathrm{A}^+} = \mathbf{u}_{\mathrm{A}^+} + [\overline{\mathrm{OB}} + \overline{\mathrm{A'B}}, 0]^{\mathrm{T}}. \tag{S5}$$

The position vector of vertex C in the deformed configuration is

$$\mathbf{x}_{\mathrm{C}} = [0, \overline{\mathrm{OB}} - v_{\mathrm{C}}]^{\mathrm{T}}, \tag{S6}$$

where v_{C} is the displacement of vertex C along the negative $y-$axis. The position vector of vertex A^- in the deformed configuration can then be expressed as

$$\mathbf{x}_{\mathrm{A}^-} = \mathbf{x}_{\mathrm{C}} + [\overline{\mathrm{AC}} * \sin(\gamma + \pi/2 - \alpha + \theta_{\mathrm{ABC}}), -\overline{\mathrm{AC}} * \cos(\gamma + \pi/2 - \alpha + \theta_{\mathrm{ABC}})]^{\mathrm{T}}, \tag{S7}$$

where θ_{ABC} is the rotation of the rigid triangle ABC.

After fully defining the deformed configuration of the metahinge architecture, we now formulate its potential energy. The strain energy of the Timoshenko beam is given by

$$U_b = \frac{E * t_0 * \overline{\mathrm{EG}}^3}{24} \int_0^{\overline{\mathrm{AG}}} (\frac{\partial \phi}{\partial \bar{y}})^2 d\bar{y} + \frac{G * t_0 * \zeta * \overline{\mathrm{EG}}}{2} \int_0^{\overline{\mathrm{AG}}} (-\phi + \frac{\partial w}{\partial \bar{y}})^2 d\bar{y}, \tag{S8}$$

where t_0 is the out-of-plane thickness (see caption of Supplementary Figure 1), E is the Young's modulus of the constituent material of the Timoshenko beam, G is the shearing modulus, and ζ is the cross-section coefficient which is selected as 0.856 [1]. We introduce an energy term $U_{\mathrm{A}^+\mathrm{A}^-}$ to penalize the relative motion between vertices A^+ and A^-, which can be expressed as

$$U_{\mathrm{A}^+\mathrm{A}^-} = \eta * \|\mathbf{x}_{\mathrm{A}^+} - \mathbf{x}_{\mathrm{A}^-}\|^2, \tag{S9}$$

where η is a sufficiently large parameter ensuring that the vertices A^+ and A^- are kinematically constrained together. Similarly, we use U_{contact} to penalize the rotation of the triangle ABC as $\theta_{\mathrm{ABC}} > \alpha$:

$$U_{\mathrm{contact}} = \sigma * H(\theta_{\mathrm{ABC}} - \alpha) * (\theta_{\mathrm{ABC}} - \alpha)^2, \tag{S10}$$

where $H(\cdot)$ is the Heaviside function, and σ is a sufficiently large parameter ensuring the hard contact behavior along the normal direction. The external work can be given by

$$W = \int_0^{v_{\mathrm{C}}} F_y dv_{\mathrm{C}}. \tag{S11}$$

Consequently, the total potential of the architecture is

$$\Pi = U_b + U_{\mathrm{A^+A^-}} + U_{\mathrm{contact}} - W. \tag{S12}$$

The equilibrium equations can then be expressed as

$$\frac{\partial \Pi}{\partial \mathbf{d}} = \mathbf{0}, \tag{S13}$$

where $\mathbf{d}$ is $[\Delta L,\ v_{\mathrm{C}},\ \theta_{\mathrm{ABC}},\ A_1,\ ...,\ A_m,\ B_1,\ ...,\ B_n]^{\mathrm{T}}$. The equilibrium path and the energy landscape are obtained via the Pseudo Acr-length method (**Algorithm 1**). The above process is implemented in an in-house Matlab code.

Algorithm 1 Pseudo Arc-length method for computing the equilibrium path of the multibody architecture

$ds \leftarrow$ the arc-length increment.
$\varepsilon \leftarrow$ the numerical tolerance.
$\mathbf{E} \leftarrow \partial\Pi/\partial\mathbf{d}$.
Let v_{C} be the control parameter for the numerical continuation.
$v_{\mathrm{C}}^{\max} \leftarrow$ the maximum value of the control parameter v_{C}.
Define symbolic vector $\mathbf{f} \leftarrow [\Delta L,\ \theta_{\mathrm{ABC}},\ A_1,\ ...,\ A_m,\ B_1,\ ...,\ B_n,\ F_y]$.
Define symbolic vector $\mathbf{s} \leftarrow [\Delta L,\ \theta_{\mathrm{ABC}},\ A_1,\ ...,\ A_m,\ B_1,\ ...,\ B_n,\ F_y,\ v_{\mathrm{C}}]$.
Let $\mathbf{s}^{\mathrm{current}}$ store the values of the symbolic vector $\mathbf{s}$; it is initially a null vector.
$\mathbf{G}_1 \leftarrow \partial\mathbf{E}/\partial\mathbf{f}$, which is a function of $\mathbf{s}$.
$\mathbf{G}_2 \leftarrow \partial\mathbf{E}/\partial v_{\mathrm{C}}$, which is a function of $\mathbf{s}$.
$t_2^{\mathrm{current}} \leftarrow \sqrt{1 + \|\mathbf{G}_1(\mathbf{s}^{\mathrm{current}})^{-1} \cdot \mathbf{G}_2(\mathbf{s}^{\mathrm{current}})\|^2}$
$\mathbf{t}_1^{\mathrm{current}} \leftarrow -t_2 * \mathbf{G}_1(\mathbf{s}^{\mathrm{current}})^{-1} \cdot \mathbf{G}_2(\mathbf{s}^{\mathrm{current}})$
$\mathbf{s}^{\mathrm{current}} \leftarrow \mathbf{s}^{\mathrm{current}} + \begin{bmatrix} \mathbf{t}_1^{\mathrm{current}} \\ t_2^{\mathrm{current}} \end{bmatrix} * ds$
$n \leftarrow$ the maximum number of iterations for each corrector step.
while $v_{\mathrm{C}} < v_{\mathrm{C}}^{\max}$ **do**
  counter $\leftarrow 1$
  $\mathbf{s}^{\mathrm{temp}} \leftarrow \mathbf{s}^{\mathrm{current}}$
  while $\|\mathbf{E}(\mathbf{s}^{\mathrm{current}})\| > \varepsilon$ **and** counter $< n$ **do** ▷ Start the corrector step
    $\mathbf{t}^{\mathrm{temp}} \leftarrow \begin{bmatrix} \mathbf{t}_1^{\mathrm{current}} \\ t_2^{\mathrm{current}} \end{bmatrix}$
    $\mathbf{s}^{\mathrm{current}} \leftarrow \mathbf{s}^{\mathrm{current}} - \begin{bmatrix} \mathbf{G}_1(\mathbf{s}^{\mathrm{current}}), & \mathbf{G}_2(\mathbf{s}^{\mathrm{current}}) \\ (\mathbf{t}_1^{\mathrm{current}})^{\mathrm{T}}, & t_2^{\mathrm{current}} \end{bmatrix}^{-1} \begin{bmatrix} \mathbf{E}(\mathbf{s}^{\mathrm{current}}) \\ (\mathbf{s}^{\mathrm{current}} - \mathbf{s}^{\mathrm{temp}})^{\mathrm{T}} \cdot \mathbf{t}^{\mathrm{temp}} - ds \end{bmatrix}$
    counter $\leftarrow$ counter+1
  end while
  $\begin{bmatrix} \mathbf{t}_1^{\mathrm{current}} \\ t_2^{\mathrm{current}} \end{bmatrix} \leftarrow \begin{bmatrix} \mathbf{G}_1(\mathbf{s}^{\mathrm{current}}), & \mathbf{G}_2(\mathbf{s}^{\mathrm{current}}) \\ (\mathbf{t}_1^{\mathrm{current}})^{\mathrm{T}}, & t_2^{\mathrm{current}} \end{bmatrix}^{-1} \cdot \begin{bmatrix} \mathbf{0} \\ 1 \end{bmatrix}$
  $\mathbf{s}^{\mathrm{current}} \leftarrow \mathbf{s}^{\mathrm{current}} + \begin{bmatrix} \mathbf{t}_1^{\mathrm{current}} \\ t_2^{\mathrm{current}} \end{bmatrix} * ds$ ▷ Compute the predictor
end while

Supplementary Note 2: Characterization of zero modes of the lattice analogy

For a two-dimensional lattice comprising N_{hinge} metahinges connected by N_{NN} nearest neighbor (NN) bonds and N_{NNN} next nearest neighbor (NNN) bonds, the infinitesimal zero modes can be obtained by examining the null space of its kinematic matrix [2]. Our focus is not on the determination of the actual elasticity of the real lattice, but rather on providing a representative assessment which we can obtain by simply assuming that all NN and NNN bonds have an axial stiffness of 1, eliminating the need to distinguish between NN and NNN bonds. The position vector of metahinge i is $\mathbf{X}_i$, and the stretching force per unit length in bond j is denoted as b_j. We define L_{ij} as

$$L_{ij} = \begin{cases} n \text{ (if metahinge } i \text{ is connected to the metahinge } n \text{ via bond } j) \\ i \text{ (if metahinge } i \text{ is not connected to any metahinges via bond } j) \end{cases} \tag{S14}$$

In the absence of any concentrated forces applied at each metahinge, the equilibrium equations for the metahinge i can be given by

$$\sum_{j=1}^{N_{\mathrm{NN}}+N_{\mathrm{NNN}}} (\mathbf{X}_i - \mathbf{X}_{L_{ij}}) * b_j = \mathbf{0}. \tag{S15}$$

By assembling the equilibrium equations of each metahinge, we can derive the equilibrium equations of the entire lattice system as

$$\mathbf{A} \cdot \mathbf{b} = \mathbf{0}, \tag{S16}$$

where $\mathbf{A}$ is the equilibrium matrix, and $\mathbf{b}$ =[b_1, ..., $b_{N_{\mathrm{NN}}+N_{\mathrm{NNN}}}$]^{T}. Next, the kinematic matrix $\mathbf{B}$ can be given by

$$\mathbf{B} = \mathbf{A}^{\mathrm{T}}. \tag{S17}$$

The null space of $\mathbf{B}$ is $[\mathbf{c}_1, ..., \mathbf{c}_{N_0+3}]$, which comprises N_0 zero modes and 3 rigid-body motions, represented by $\mathbf{d}_i (i = 1, 2, 3)$. To extract the zero modes (internal mechanisms) from the null space, we apply the following orthogonalization

$$\mathbf{c}_i = \mathbf{c}_i - \frac{\mathbf{c}_i \cdot \mathbf{d}_j}{\|\mathbf{d}_j\|^2} \cdot \mathbf{d}_j. \tag{S18}$$

The remaining non-zero $\mathbf{c}_i$ constitutes the zero modes (internal mechanisms) of the lattice analogy.

Supplementary Note 3: Nonlinear Finite Element simulation of biaxial zero modes

To computationally investigate the biaxial zero modes illustrated in Fig.3c, 3d, and 3e, we resorted to Abaqus 2020 (Dassault Systèmes) and created a Finite Element (FE) model comprising truss elements (T2D2) and axial connector elements (CONN2D2), which represent the nearest neighbor (NN) bonds and the next nearest neighbor (NNN) bonds respectively. Both the truss elements and connectors have an axial stiffness of 1. The deformation response is computed using the General Static solver with geometric nonlinearity. The lattice FE model, boundary conditions, and the corresponding physical specimens are illustrated in Supplementary Figure 2, where distinct colors are used to highlight the areas enclosed by the rotational triangles. In the FE model, the rotation angle of each triangle is evaluated as the average rotation angle of its three constituent edges. By comparing each enclosed area of the FE model and the physical experiment specimen, we can observe a good agreement, a result that validates that the deactivation of a metahinge is equivalent to adding a pair of NNN bonds to its adjacent rotation triangles.

Leveraging the FE model, we obtained the force-displacement relation as well as the evolution of the incremental stiffness versus the displacement, as shown in Supplementary Figure 3. The initial incremental stiffness in all three cases is zero but becomes positive as the displacement increases due to geometric nonlinearity, confirming that the corresponding biaxial zero mode is infinitesimal. The lattice models in Supplementary Figure 3a and 3c manifest a stiffening behavior as the applied load amplifies; the lattice in Supplementary Figure 3b, however, exhibits a softening behavior once u_1/l surpasses approximately 0.45. Comparing their incremental stiffness at a modest deformation level, e.g., when $u_1/l \approx 0.2$, it can be observed that the lattice model showing the smallest modal amplitude along the $\mathbf{e}_1$ axis (Supplementary Figure 2a) has the highest stiffness (Supplementary Figure 3a), whereas the lattice model exhibiting the largest modal amplitude along the $\mathbf{e}_1$ axis (Supplementary Figure 2c) is the softest in response to the applied force F_1 (Supplementary Figure 3c).

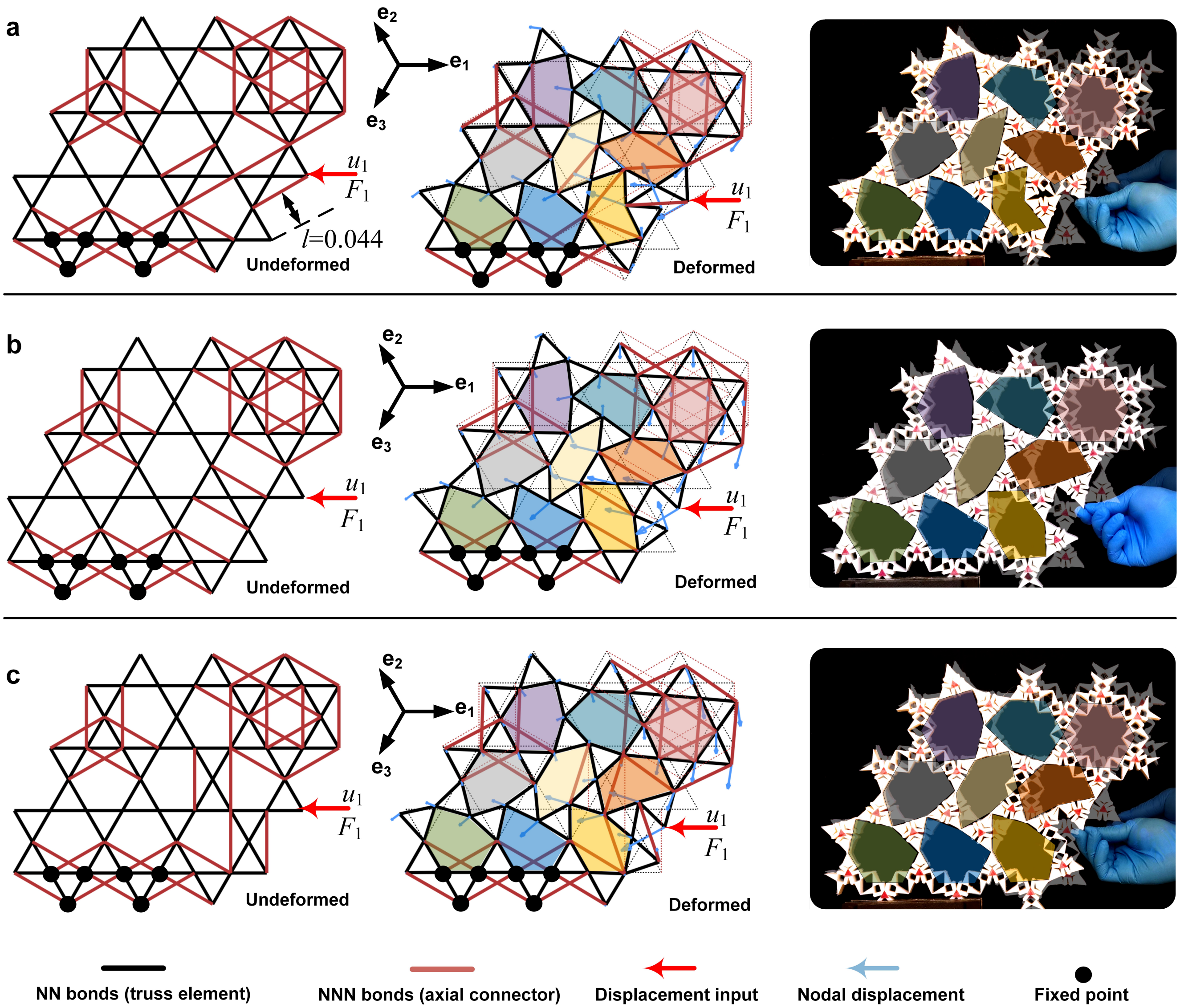

Supplementary Figure 2. **Nonlinear FE simulations of the lattice analogs bearing biaxial zero modes. a, b, c,** FE models for the selectively activated Kagome lattices presented in Fig.3c, 3d, and 3e; **a,** the lattice exhibits a larger modal amplitude along the $\mathbf{e}_2$ axis. **b,** the lattice has equivalent modal amplitudes along the $\mathbf{e}_1$ and $\mathbf{e}_2$ axes. **c,** the lattice manifests a larger modal amplitude along the $\mathbf{e}_1$ axis; the areas enclosed by the rotational triangles are shaded in distinct colors for better comparison between the FE and experimental results.

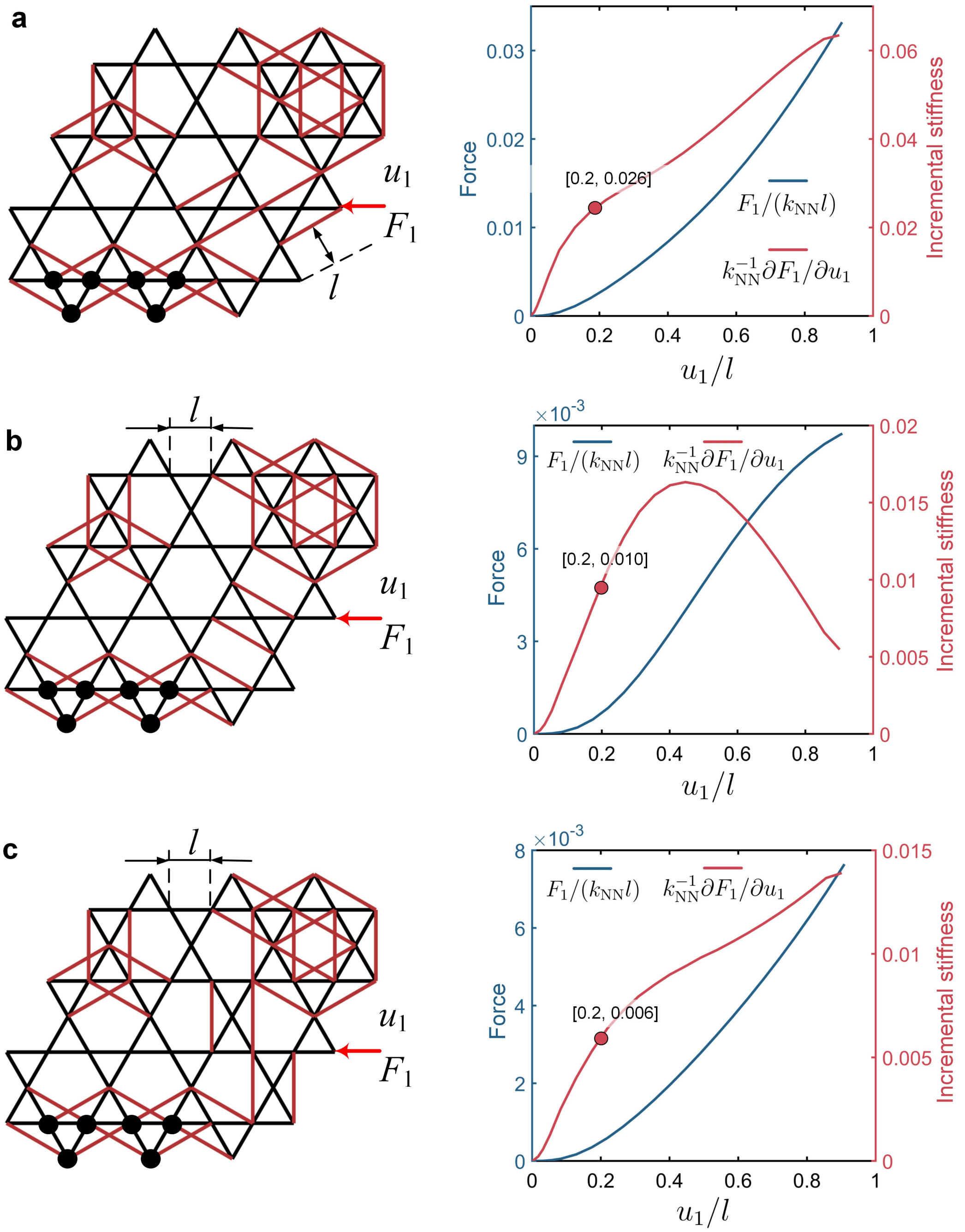

Supplementary Figure 3. **Force-displacement and stiffness-displacement relations of lattice models bearing infinitesimal biaxial zero modes. a, b, c,** the lattice models exhibit zero stiffness along the $\mathbf{e}_1$ axis in their initial configurations while delivering a positive stiffness in the finite-deformation regime.

Supplementary Note 4: Minimum energy path of the periodic Kagome lattice with selectively added NNN bonds

The periodic Kagome lattice with selectively added NNN bonds is shown on the left of Supplementary Figure 4, where its primitive unit cell is highlighted in yellow with an initial area of A. The nonlinear deformation of the periodic lattice exhibits translational symmetry, and hence we can extract its unit cell for analyzing the minimum energy path (MEP) during state transition. As illustrated in the middle and right of Supplementary Figure 4, the unit cell comprises independent metahinges (red), dependent metahinges (green), independent NN bonds (outlined in blue), and independent NNN bonds (outlined in red); the motion of dependent metahinges is dependent on the corresponding independent metahinges thanks to the translational symmetry.

The initial coordinates of each metahinge are defined in the Cartesian coordinate system $x - o - y$, and denoted by $\mathbf{X}_i$, where the subscript i is the label of each metahinge. The displacement and deformed position of each meta-hinge are $\mathbf{u}_i$ and $\mathbf{x}_i$ respectively, and hence we have

$$\mathbf{x}_i = \mathbf{X}_i + \mathbf{u}_i. \tag{S19}$$

To eliminate macroscopic rigid-body motions, we assume that the metahinge 5 is fixed at the origin point, and the metahinge 15 is only allowed to slide along the $y-$axis. We use a 15×15 matrix $\mathbf{C}^{\mathrm{NN}}$ to store the connectivity information of each NN bond and $\mathbf{C}^{\mathrm{NNN}}$ for each NNN bond. For example, as the metahinge 1 is connected to the metahinge 4 via an NN bond, the elements $C_{1,4}^{\mathrm{NN}}$ and $C_{4,1}^{\mathrm{NN}}$ have a value of 1; given metahinge 1 is not connected to metahinge 2 via neither an NN bond nor an NNN bond, the elements $C_{1,2}^{\mathrm{NN}}$, $C_{2,1}^{\mathrm{NN}}$, $C_{1,2}^{\mathrm{NNN}}$, and $C_{2,1}^{\mathrm{NNN}}$ are 0. The total strain energy of the unit cell U_{total} incorporates the contribution of NN bonds and that of NNN bonds, and can be expressed as

$$\begin{aligned} U_{\mathrm{total}} = &\sum_{i=1,j=i}^{15,15} \frac{k_{\mathrm{NN}} * C_{i,j}^{\mathrm{NN}}}{2} * (\|\mathbf{x}_i - \mathbf{x}_j\| - \|\mathbf{X}_i - \mathbf{X}_j\|)^2 + \\ &\sum_{i=1,j=i}^{15,15} \frac{k_{\mathrm{NNN}} * C_{i,j}^{\mathrm{NNN}}}{2} * (\|\mathbf{x}_i - \mathbf{x}_j\| - \|\mathbf{X}_i - \mathbf{X}_j\|)^2. \end{aligned} \tag{S20}$$

The translational symmetry imposes kinematic constraints between independent metahinges and dependent metahinges as

$$\begin{aligned}\mathbf{u}_1 &= \mathbf{u}_{12} - \mathbf{u}_{15},\\ \mathbf{u}_3 &= \mathbf{u}_{14} - \mathbf{u}_{15},\\ \mathbf{u}_8 &= \mathbf{u}_{10} + \mathbf{u}_2.\end{aligned} \tag{S21}$$

Substituting Eq. S21 to Eq. S20 yields the strain energy formulation expressed by independent degrees of freedom. The state of the unit cell at an intermediate moment i is characterized by $\mathbf{q}^i$ =[$\mathbf{u}_2^i$, $\mathbf{u}_4^i$, ..., $\mathbf{u}_7^i$, $\mathbf{u}_9^i$, ..., $\mathbf{u}_{15}^i$]. We also note that the intersecting/contact between bonds is not taken into account in this lattice analogy, but the intersecting/contact might arise in a physical metamaterial specimen, affecting the multistable transition behavior, as shown in Fig. 4f.

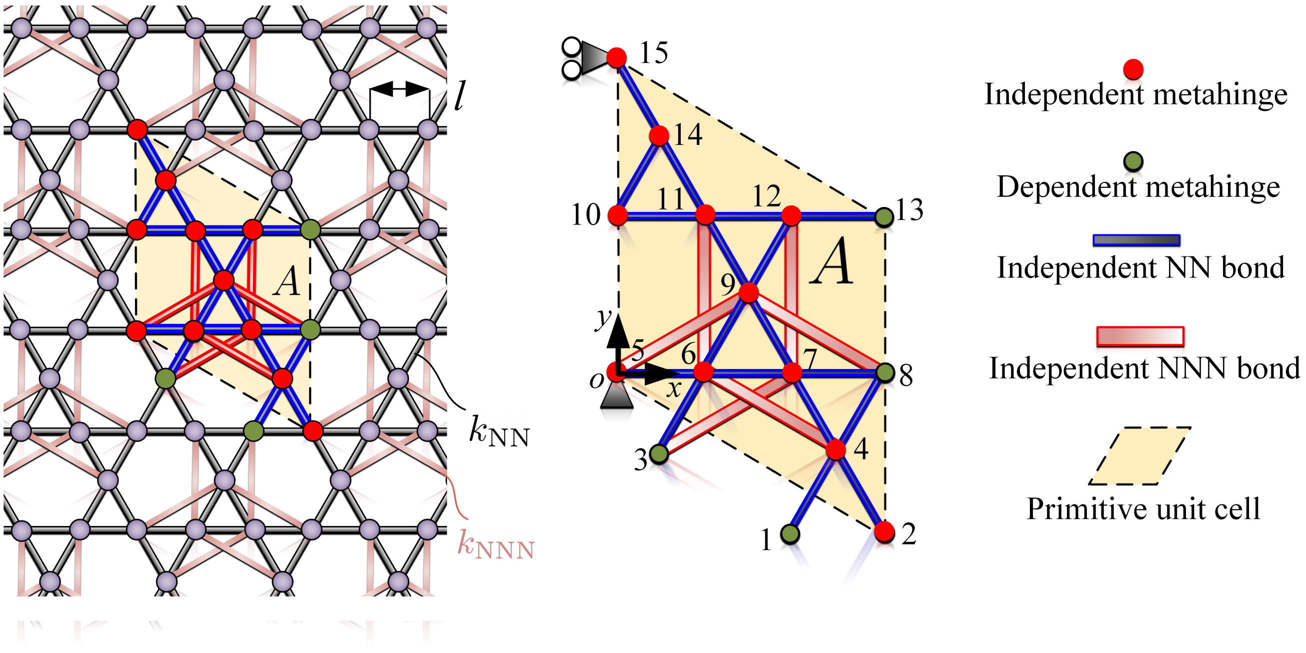

Supplementary Figure 4. **Periodic Kagome lattice with selectively added NNN bonds resembling the deployed state of the multistable auxetic kirigami sheet with triangular motifs [3].** The periodic lattice can be tessellated by a unit cell comprising independent metahinges, NN bonds, and NNN bonds.

The Nudged Elastic Band (NEB) method is a numerical algorithm used to predict the MEP connecting two local energy minimum points [4–7], i.e. two stable states of a bistable mechanical system. The NEB method is based on conventional gradient-based optimization algorithms, and

its variables are an augmented vector containing the degrees of freedom of the system across all its intermediate states, i.e. $[\mathbf{q}^2, ..., \mathbf{q}^i, ..., \mathbf{q}^{I-1}]$, where I is the total number of states; $\mathbf{q}^1$ is the initial displacement of the unit cell, which is a null vector; $\mathbf{q}^I$ is the final displacement of the unit cell, corresponding to the displacement field in the fully collapsed state. The final displacement of each independent metahinge can be given by

$$\begin{aligned}
&\mathbf{u}_3^I = \mathbf{u}_4^I = \mathbf{u}_5^I = \mathbf{u}_6^I = \mathbf{u}_7^I = \mathbf{u}_8^I = \mathbf{u}_9^I = \mathbf{u}_{11}^I = \mathbf{u}_{12}^I = \mathbf{0},\\
&\mathbf{u}_1^I = [0, \sqrt{3}l],\ \mathbf{u}_2^I = [-\frac{3l}{2}, \frac{\sqrt{3}l}{2}],\ \mathbf{u}_{10}^I = [\frac{3l}{2}, -\frac{\sqrt{3}l}{2}],\\
&\mathbf{u}_{13}^I = [-\frac{3l}{2}, -\frac{\sqrt{3}l}{2}],\ \mathbf{u}_{14}^I = [0, -\sqrt{3}l],\ \mathbf{u}_{15}^I = [0, -\sqrt{3}l].
\end{aligned} \tag{S22}$$

The initial guess of the MEP is a linear interpolation between $\mathbf{q}^1$ and $\mathbf{q}^I$, and hence $\mathbf{q}^i$ is given by

$$\mathbf{q}^i = \mathbf{q}^1 + \frac{(\mathbf{q}^I - \mathbf{q}^1) * (i-1)}{I-1},\ (i = 2, ..., I-1). \tag{S23}$$

After initializing the MEP, we can iteratively update the MEP utilizing **Algorithm 2**, **3**, and **4**. Through **Algorithm 2**, we evaluate the searching direction $\mathbf{s}_{\text{current}}$ at each iteration step through the conjugate method; the maximum number of iterations $n_{\max}$, scale factor α_0, and tolerance of the driving force ε are 2×10^5, 0.05, and 10^{-6}. In the conjugate method, the search direction at the current iteration $\mathbf{s}_{\text{current}}$ is a linear combination of the current driving force $\mathbf{f}_{\text{current}}$ and the searching direction in the previous iteration $\mathbf{s}_{\text{previous}}$. In **Algorithm 3**, we evaluate the driving force at each intermediate state; the driving force at each intermediate state is a sum of the realistic force perpendicular to the tangent of the transition path and a fictitious force generated by the elastic band; the tangential stiffness of the fictitious elastic band k_{band} is 10. Through **Algorithm 4**, we evaluate the tangential vector of the elastic band at each intermediate state point; this tangential vector is used in **Algorithm 3** to evaluate the driving force.

Supplementary Figure 5 illustrates the convergence of the norm of the driving force, i.e. $\|\mathbf{f}_{\text{current}}\|$ (see **Algorithm 1**) versus the number of iterations. At the beginning of the optimization process, it shows a superlinear rate of convergence followed by a sublinear convergence behavior; as the number of iterations becomes substantial, the rate of convergence becomes linear.

Algorithm 2 Iteratively update the MEP

Given the initial guess of the MEP $[\mathbf{q}^2, ..., \mathbf{q}^i, ..., \mathbf{q}^{I-1}]$, we first evaluate the driving force $\mathbf{f}_{\text{current}}$ at the intermediate state points (see more details in **Algorithm 3** to know how to evaluate the driving forces).

$\mathbf{f}_{\text{current}} \leftarrow [\text{Force}(\mathbf{q}^2), ..., \text{Force}(\mathbf{q}^i), ..., \text{Force}(\mathbf{q}^{I-1})]$
$n_{\max} \leftarrow$ the maximum number of iterations.
$\alpha_0 \leftarrow$ scale factor
$\varepsilon \leftarrow$ tolerance of the driving force
for counter$\leftarrow$1 to $n_{\max}$ **do**
  if counter > 1 **then**
    if $\|\mathbf{f}_{\text{current}}\| < \varepsilon$ **then**
      return $[\mathbf{q}^2, ..., \mathbf{q}^i, ..., \mathbf{q}^{I-1}]$
      break
    end if
    if $\mathbf{f}_{\text{current}} \cdot \mathbf{f}_{\text{current}}^{\text{T}} \neq 0$ **then**
      $\beta \leftarrow (\mathbf{f}_{\text{current}} \cdot \mathbf{f}_{\text{current}}^{\text{T}})/(\mathbf{f}_{\text{previous}} \cdot \mathbf{f}_{\text{previous}}^{\text{T}})$
    else
      $\beta \leftarrow 1$
    end if
    $\mathbf{s}_{\text{current}} \leftarrow \mathbf{f}_{\text{current}} + \mathbf{s}_{\text{previous}} * \beta$
  else
    $\mathbf{s}_{\text{current}} \leftarrow \mathbf{f}_{\text{current}}$
  end if
  if $\|\mathbf{s}_{\text{current}}\| > 1$ **then**
    $\mathbf{s}_{\text{current}} \leftarrow \mathbf{s}_{\text{current}}/\|\mathbf{s}_{\text{current}}\|$
  end if
  $\mathbf{s}_{\text{current}} \leftarrow \mathbf{s}_{\text{current}} * \alpha_0$
  $\mathbf{s}_{\text{previous}} \leftarrow \mathbf{s}_{\text{current}}$
  $\mathbf{f}_{\text{previous}} \leftarrow \mathbf{f}_{\text{current}}$
  $[\mathbf{q}^2, ..., \mathbf{q}^i, ..., \mathbf{q}^{I-1}] \leftarrow [\mathbf{q}^2, ..., \mathbf{q}^i, ..., \mathbf{q}^{I-1}]+\mathbf{s}_{\text{current}}$
  $\mathbf{f}_{\text{current}} \leftarrow [\text{Force}(\mathbf{q}^2), ..., \text{Force}(\mathbf{q}^i), ..., \text{Force}(\mathbf{q}^{I-1})]$
end for
return $[\mathbf{q}^2, ..., \mathbf{q}^i, ..., \mathbf{q}^{I-1}]$

Algorithm 3 Evaluate the driving force at state point $\mathbf{q}^i$: Force($\mathbf{q}^i$)

To evaluate the driving force at intermediate state $\mathbf{q}^i$, we need to evaluate the gradient of the potential energy at this point.
$\mathbf{F}_{\text{strain}} \leftarrow -\nabla U_{\text{total}}(\mathbf{q}^i)$
Also, we need to evaluate the tangent at state point $\mathbf{q}^i$ (see **Algorithm 3** for details).
$\mathbf{t} \leftarrow \text{Tangent}(\mathbf{q}^i)$.
The projection of $\mathbf{F}_{\text{strain}}$ with respect to $\mathbf{t}$ is $\mathbf{F}_{\text{strain}||}$.
$\mathbf{F}_{\text{strain}||} \leftarrow \mathbf{F}_{\text{strain}} \cdot \mathbf{t}$
The orthogonal component of $\mathbf{F}_{\text{strain}}$ is $\mathbf{F}_{\text{strain}\perp}$.
$\mathbf{F}_{\text{strain}\perp} \leftarrow \mathbf{F}_{\text{strain}} - \mathbf{F}_{\text{strain}||}$
We assume that there exists a fictitious elastic band connecting a sequence of intermediate states. This fictitious elastic band can prevent each state point from converging to the local energy minimum points during iterations. This fictitious elastic band has a tangential stiffness of k_{band}. The driving force generated by the elastic band is $\mathbf{F}_{\text{band}}$.
$k_{\text{band}} \leftarrow$ the tangential stiffness of the fictitious elastic band.
$\mathbf{F}_{\text{band}} \leftarrow k_{\text{band}} * \|\mathbf{q}^{i+1} - \mathbf{q}^i\| \cdot \mathbf{t} - k_{\text{band}} * \|\mathbf{q}^i - \mathbf{q}^{i-1}\| \cdot \mathbf{t}$
The total driving force comprises $\mathbf{F}_{\text{strain}\perp}$ and $\mathbf{F}_{\text{band}}$.
$\mathbf{F}_{\text{total}} \leftarrow \mathbf{F}_{\text{strain}\perp} + \mathbf{F}_{\text{band}}$
return $\mathbf{F}_{\text{total}}$

Algorithm 4 Evaluate the tangent vector at state point $\mathbf{q}^i$: Tangent($\mathbf{q}^i$)

To evaluate the tangent at state point $\mathbf{q}^i$, we also need to know the energy information of its two adjacent state points $\mathbf{q}^{i-1}$ and $\mathbf{q}^{i+1}$.

```
U_1 ← U_total(q^{i-1})
U_2 ← U_total(q^i)
U_3 ← U_total(q^{i+1})
a_max ← max(|U_3 − U_2|, |U_2 − U_1|)
a_min ← min(|U_3 − U_2|, |U_2 − U_1|)
if U_3 ≥ U_2 ≥ U_1 then
    t ← (q^{i+1} − q^i)
else
    if U_3 ≤ U_2 ≤ U_1 then
        t ← (q^i − q^{i-1})
    else
        if U_3 > U_1 and (U_2 ≥ U_3 or U_2 ≤ U_1) then
            t ← (q^{i+1} − q^i) * a_max + (q^i − q^{i-1}) * a_min
        else
            if U_3 ≤ U_1 and (U_2 ≤ U_3 or U_2 ≥ U_1) then
                t ← (q^{i+1} − q^i) * a_min + (q^i − q^{i-1}) * a_max
            end if
        end if
    end if
end if
t ← t/‖t‖
return t
```

In Supplementary Figure 6, we demonstrate representative intermediate states of the periodic lattice transitioning along the MEP; the symmetry of the lattice is well preserved during state transition.

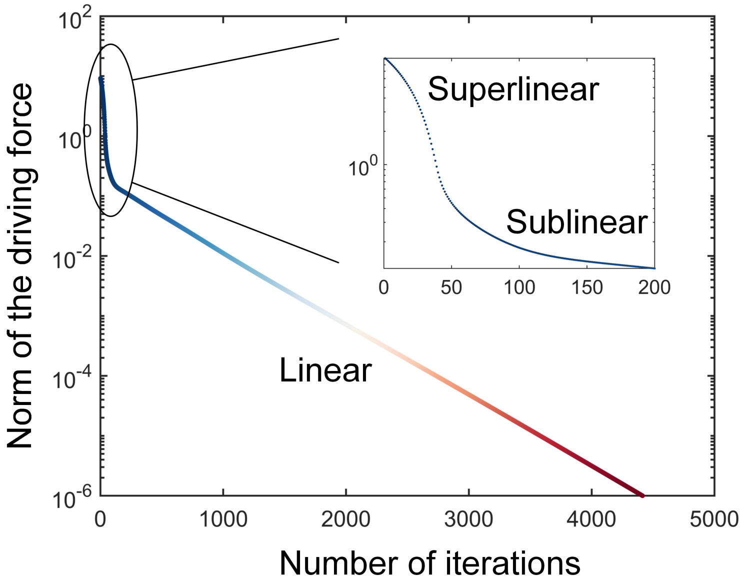

Supplementary Figure 5. **Convergence of the norm of driving force with respect to the number of iterations.** The convergence criterion is satisfied after 4414 iterations.

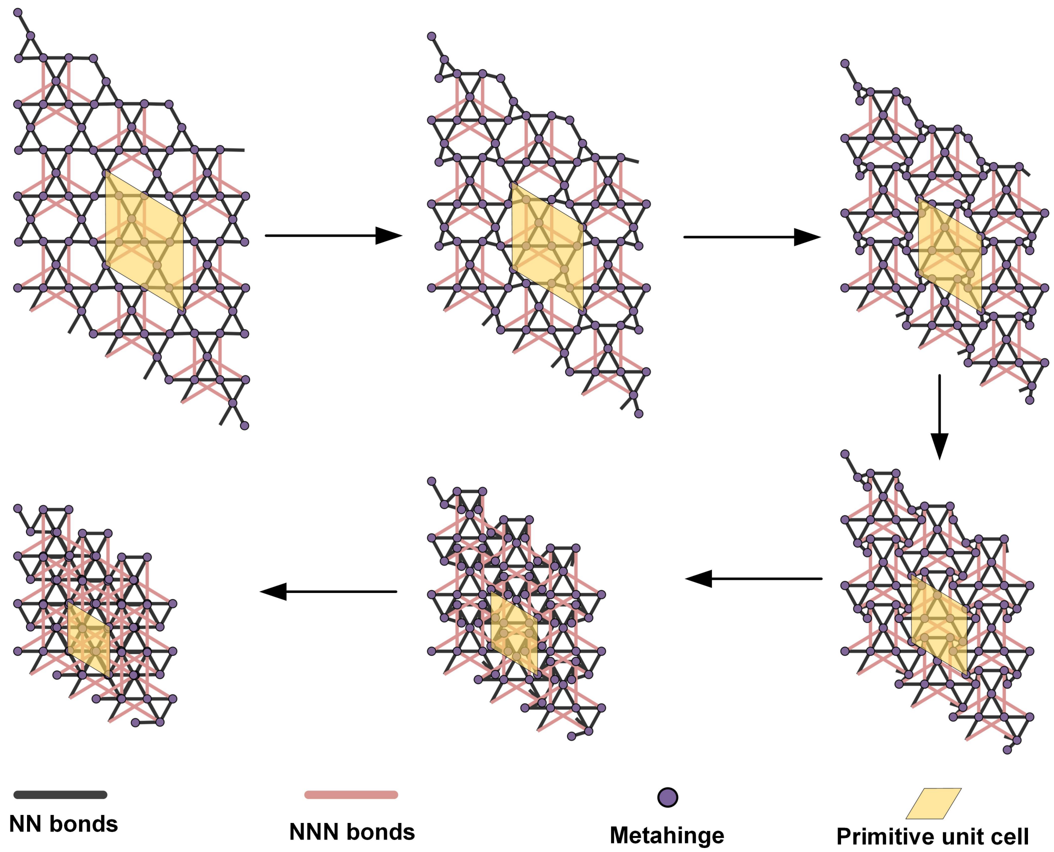

Supplementary Figure 6. **Intermediate states of the periodic lattice transitioning along the MEP.** The area of the primitive unit cell in the fully collapsed state shrinks to 25% of its initial value.

Supplementary Note 5: Phonon spectrum of the periodic Kagome lattice with selectively added NNN bonds

We choose the periodic lattice presented in Fig.4h as a representative example to demonstrate the computation of the phonon spectrum of a Kagome lattice with selectively added NNN bonds. The unit cell is shaded in yellow, and the lattice vectors in direct space are $\mathbf{a}_1$ and $\mathbf{a}_2$, as shown in Supplementary Figure 7. Given the translation symmetry of the periodic lattice, there exist two types of metahinges, which are independent metahinges marked in red and dependent metahinges in green. The displacement of a dependent metahinge can be represented by the displacement of its corresponding independent metahinge via the Bloch's theorem. In particular, in the initial configuration, the position vector of dependent metahinge i, $\mathbf{X}_i^{\text{dependent}}$, can be expressed as

$$\mathbf{X}_i^{\text{dependent}} = \mathbf{X}_i^{\text{independent}} + n_{1,i}\mathbf{a}_1 + n_{2,i}\mathbf{a}_2, \tag{S24}$$

where $\mathbf{X}_i^{\text{independent}}$ is the position vector of the independent metahinge that can be translated from $\mathbf{X}_i^{\text{dependent}}$ through vector $-n_{1,i}\mathbf{a}_1 - n_{2,i}\mathbf{a}_2$, where $n_{1,i}$ and $n_{2,i}$ are integers; the $(n_{1,i}, n_{2,i})$ for each dependent metahinge is unique. Through the Bloch's theorem, the displacement of the dependent metahinge i, $\mathbf{u}_i^{\text{dependent}}$, can be expressed as

$$\mathbf{u}_i^{\text{dependent}} = \mathbf{u}_i^{\text{independent}} e^{j\mathbf{k}\cdot(n_{1,i}\mathbf{a}_1 + n_{2,i}\mathbf{a}_2)}, \tag{S25}$$

where $j = \sqrt{-1}$, and $\mathbf{k} = [k_x, k_y]$ is the wave vector. In the following, we drop out the superscript "independent" or "dependent" for simplicity. The governing equations of the independent metahinge i is given by

$$-m_0\mathbf{u}_{i,tt} + \sum_{n=1}^{n_{\text{NN}}} k_{\text{NN}}\mathbf{t}_{ni}^{\text{T}} \cdot (\mathbf{u}_n - \mathbf{u}_i) \cdot \mathbf{t}_{ni} + \sum_{s=1}^{n_{\text{NNN}}} k_{\text{NNN}}\mathbf{t}_{si}^{\text{T}} \cdot (\mathbf{u}_s - \mathbf{u}_i) \cdot \mathbf{t}_{si} = \mathbf{0}, \tag{S26}$$

where $u_{i,tt}$ denotes the second derivative of u_i with respect to time, $\mathbf{u}_n$ is the displacement of the metahinge that is connected to metahinge i via an NN bond, n_{NN} is the total number of metahinges that are connected to metahinge i via an NN bond, $\mathbf{u}_s$ is the displacement of the metahinge that

is connected to metahinge i via an NNN bond, n_{NNN} is the total number of metahinges that are connected to metahinge i via an NNN bond, and $\mathbf{t}_{ni}$ or $\mathbf{t}_{si}$ is a unit vector pointing from metahinge i to metahinge n or s. We then substitute Eq. S25 into Eq. S26 to replace all the dependent displacement vectors with independent displacement vectors and assume that the steady response is harmonic, i.e. $\mathbf{u}_i = \bar{\mathbf{u}}_i e^{j\omega t}$. As a result, the governing equations for the entire lattice model can be succinctly formulated as

$$(\mathbf{K} - \omega^2 \mathbf{M})\bar{\mathbf{u}} = \mathbf{0}, \tag{S27}$$

where $\mathbf{K}$ is the stiffness matrix as a function of the wave vector $[k_x, k_y]$, $\mathbf{M}$ denotes the mass matrix, which is an identity matrix under the assumption of isotropic unit masses for all metahinges; $\bar{\mathbf{u}}$ is an augmented vector encompassing the amplitudes of all independent displacement vectors. By solving the following eigenvalue problem

$$\det(\mathbf{K} - \omega^2 \mathbf{M}) = 0 \tag{S28}$$

across varying wave vectors in the first Brillouin Zone, we obtain the phonon spectrum, i.e. the relationship between ω and $\mathbf{k}$.

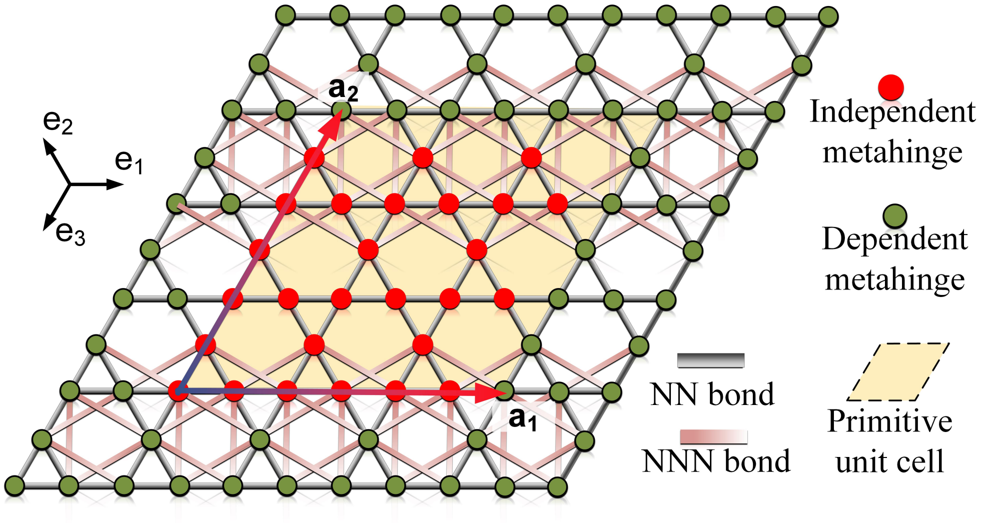

Supplementary Figure 7. Periodic Kagome lattice with selectively added NNN bonds allowing a zero mode to travel along the $\mathbf{e}_1$ axis. The displacement of dependent metahinges is a function of the displacement of dependent metahinges and wave vector.

In addition to examining the $\omega - \mathbf{k}$ relation of the periodic lattices illustrated in Fig.4h, 4i, and 4j, we also investigated their phase velocity close to the $\mathbf{k} = [0, 0]$ point in reciprocal space. To do so, we let $\mathbf{k} = \delta[\sin\theta, \cos\theta]$, where δ is a sufficiently small number. Then, we assessed $c_p = \|\omega_1/\delta\|$ [8] within the range $\theta \in [0, 2\pi]$, where ω_1 is the frequency of the first acoustic branch. In Supplementary Figure 8, we illustrate the normalized phase velocity $c_p/\sqrt{k_{\mathrm{NN}} l^2/m_0}$ as a function of θ. For the lattice shown in Supplementary Figure 8a, it has zero phase velocity at $\theta = 90°$; the lattice in Supplementary Figure 8b manifests zero phase velocity at $\theta = 90°$ and $\theta = 150°$; the low-frequency dynamic characteristics of both lattices exhibit pronounced sensitivity to the direction of the wave vector, highlighting a strong anisotropy around the $\mathbf{k} = [0, 0]$ point. The lattice tessellated by the unit cell bearing a biaxial ZM (Supplementary Figure 8c), however, has a milder anisotropy around the $\mathbf{k} = [0, 0]$ point; the minimum phase velocity is non-zero and occurs at the angle bisector of $\theta = 90°$ and $\theta = 150°$, i.e. $\theta = 120°$. This result implies that by adding NNN bonds, we can also reprogram the degree of anisotropy of the Kagome metamaterial.

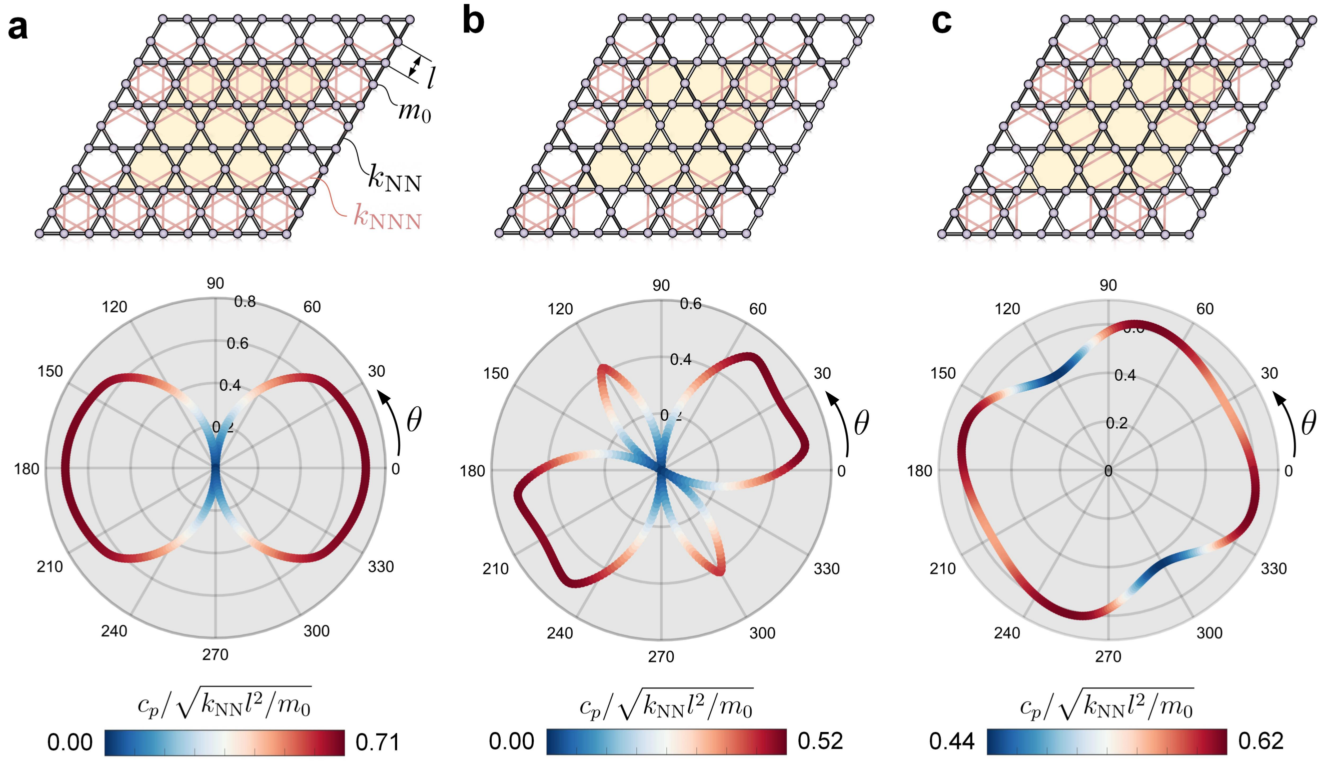

Supplementary Figure 8. **Phase velocity of the Kagome lattice with selectively added NNN bonds as a function of the direction of the wave vector** $\boldsymbol{\theta}$**. a, b, c,** The lattice models exhibit distinct anisotropic behaviors close to the $[k_x,\ k_y] = [0.0,\ 0.0]$ point in reciprocal space; the lattice tessellated by the unit cell bearing a biaxial zero mode (**c**) has the mildest anisotropy in the low-frequency regime.

Supplementary Note 6: Phonon spectrum evolution of the lattice exhibiting non-isochoric reconfiguration

The metamaterials illustrated in Fig.6 exhibit a non-isochoric transformation. As a result, the activation/deactivation of their lattice analogs is no longer equivalent to the deletion/addition of the NNN bonds; rather, it is equivalent to diminishing the aspect ratio of the four-node-five-bond rectangle, s_1/s_2, to zero such that pairing hinges coincide, and the two-dimensional rectangle degenerates into a one-dimensional bond. In the deactivated state, i.e. the initial state, the aspect ratio s_1/s_2 is equal to $\overline{\mathrm{OC}}/L = 0.64$ (see Supplementary Figure 1 for $\overline{\mathrm{OC}}$ and L). We compute the phonon spectrum of the lattice with $s_1/s_2 = 0.64$ (initial deactivated state), $s_1/s_2 = 0.4$, $s_1/s_2 = 0.2$, and $s_1/s_2 = 0.001$ (activated state) to demonstrate how the band structure evolves thus allowing band gaps to emerge or vanish, and how the zero-frequency mode generates.

The *p6mm* lattice in Supplementary Figure 9a with $s_1/s_2 = 0.64$ (initial deactivated state) has a bandgap between the fourth and fifth branches, which is shaded in grey. As s_1/s_2 gradually approaches 0, the grey bandgap is broadened, and the branches from the fifth to the ninth converge to a flat band, opening up a bandgap between the ninth and tenth branches (blue area). The phase velocity of the first branch approaches zero, i.e., a zero-frequency mode emerges along the contour of the irreducible Brillouin Zone.

The *p4mm* lattice depicted in Supplementary Figure 9b has a narrow bandgap between the sixth and seventh branches (blue area) when $s_1/s_2 = 0.64$, and the fourth and fifth branches intersect. As s_1/s_2 decreases to 0.4, this intersection is eliminated; a bandgap shaded in grey emerges between these two branches when s_1/s_2 further reduces to 0.2. As s_1/s_2 approaches 0, the first branch degenerates to a zero-frequency mode along the Γ-Y contour, the third and fourth branches merge into a flat band, as do the fifth and sixth branches, and the originally existing blue bandgap disappears.

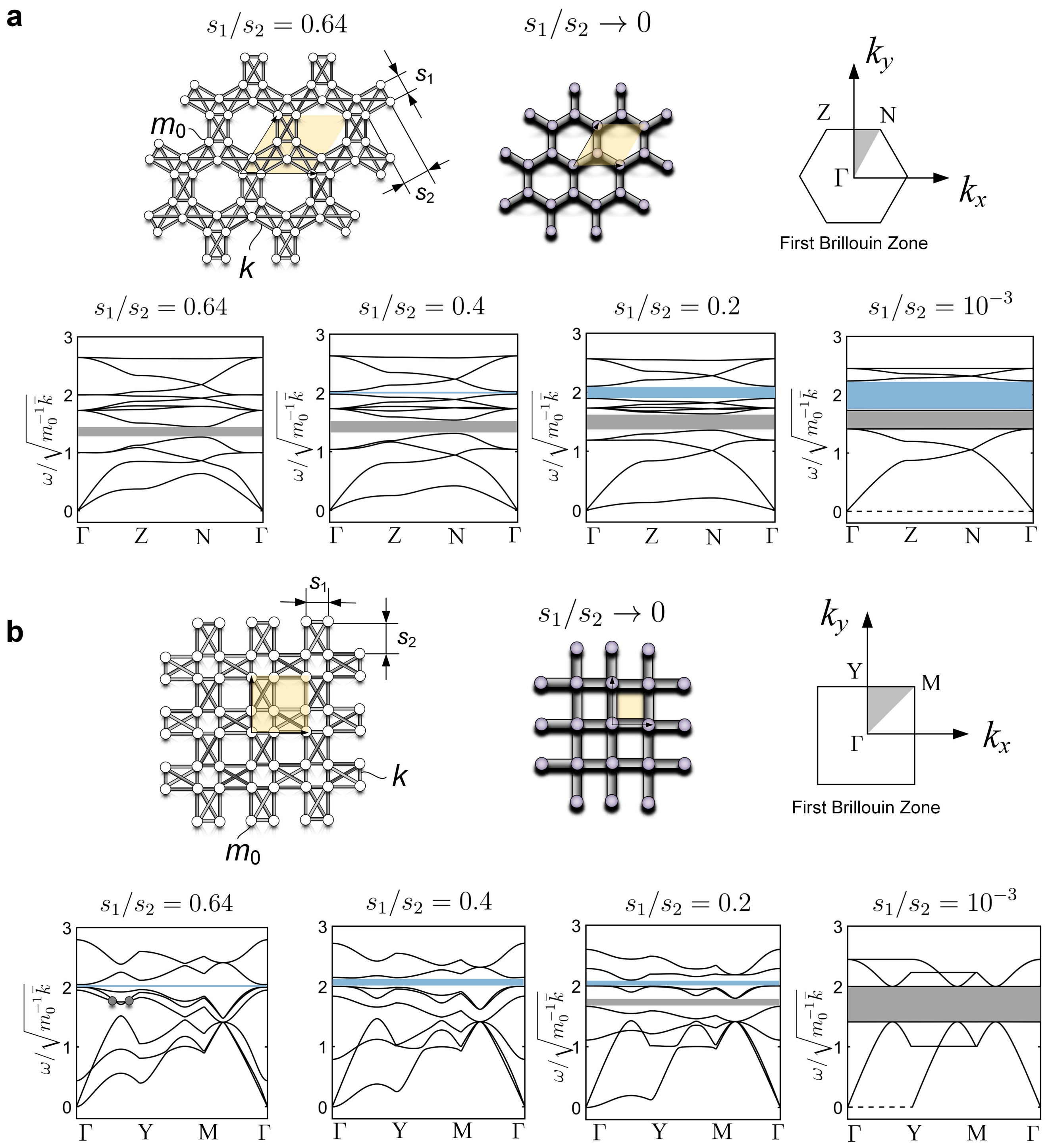

Supplementary Figure 9. **Evolution of the phonon spectra of the lattice analogs showing non-isochoric reconfiguration. a,** The *p6mm* lattice analogy and its phonon spectra. **b,** The *p4mm* lattice analogy and its phonon spectra.

Supplementary Note 7: Geometry of experimental specimens

The geometry of the metahinge specimen shown in Fig.1f originates from the theoretical model illustrated in Supplementary Figure 1; due to the non-negligible size of the flexural hinge, there exists a slight geometric difference between them, as demonstrated in Supplementary Figure 10. This architecture is then connected in a certain manner to build up metamaterial specimens demonstrated in Fig.2, 3, 4, and 5.

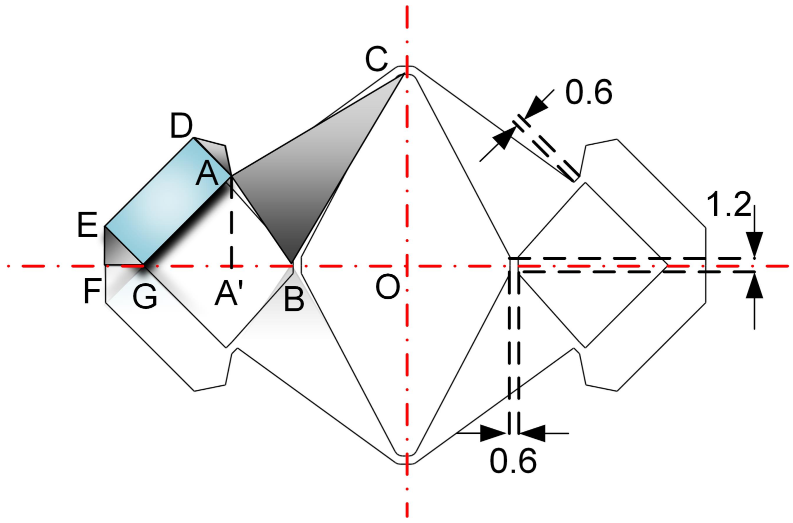

Supplementary Figure 10. **Geometric relationship between the theoretical model illustrated in Supplementary Figure 1 and the bistable metahinge architecture used for fabrication.** The fillet at hinge C has a radius of 0.6 mm; the length dimensions of the multi-body model and the bistable architecture for fabrication are consistent. Unit is mm.

The finite-period metamaterial sample shown in Fig.2 and Fig.3 was assembled by multiple pieces of 3D-printed sub-parts via interlocking connection and then reinforced by adhesion, as illustrated in Supplementary Figure 11a. The metamaterial sample shown in Fig.5 was integrally printed without any additional connections. The metamaterial samples manifesting non-isochoric reconfiguration (Fig.6) were also assembled by multiple pieces of additively manufactured sub-parts; the corresponding assembly schematic diagram is shown in Supplementary Figure 11b and c. Other 3D printing processes, for example, Selective Laser Sintering and Multi Jet Fusion, can also be used to fabricate the metamaterial sample capable of reversible large deformation.

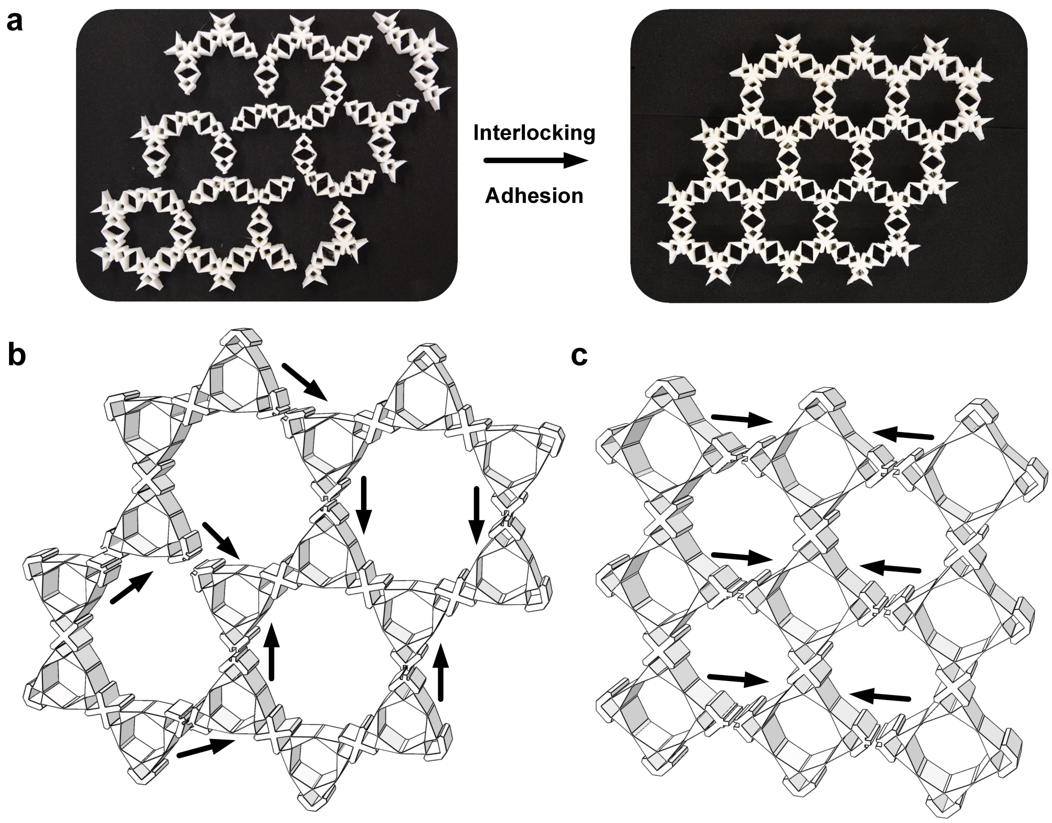

Supplementary Figure 11. **Assembly schematic diagram of finite-period metamaterial specimens. a,** Assembly of the Kagome-type metamaterial. **b, c,** Assembly of the metamaterials bearing metahinges with a coordination number of three and four; black arrows indicate how to connect these sub-parts.

Supplementary Note 8: Supplementary Movies

Supplementary Movie 1: Activation and deactivation of the metahinge.

Supplementary Movie 2: Isochoric reconfiguration of the Kagome-type metamaterial capable of substantial stiffness reprogramming.

Supplementary Movie 3: Harnessing biaxial zero modes in a selectively activated Kagome metamaterial to transmit mechanical signals.

Supplementary Movie 4: Achieving mechanical logic operations through the biaxial zero mode.

Supplementary Movie 5: Multistable transition of a selectively activated Kagome metamaterial.

Supplementary Movie 6: Reprogramming the buckling mode in a rotation-square metamaterial via selective activation of metahinges.

Supplementary Movie 7: Non-isochoric reconfiguration of the metamaterial bearing metahinges with a coordination number of three.

Supplementary Movie 8: Non-isochoric reconfiguration of the metamaterial bearing metahinges with a coordination number of four.

References

[1] Lei Wu and Damiano Pasini. In-situ activation of snap-through instability in multi-response metamaterials through multistable topological transformation. *Advanced Materials*, 2301109, 2023.

[2] Sergio Pellegrino and Christopher Reuben Calladine. Matrix analysis of statically and kinematically indeterminate frameworks. *International Journal of Solids and Structures*, 22(4):409–428, 1986.

[3] Ahmad Rafsanjani and Damiano Pasini. Bistable auxetic mechanical metamaterials inspired by ancient geometric motifs. *Extreme Mechanics Letters*, 9:291–296, 2016.

[4] Graeme Henkelman, Blas P Uberuaga, and Hannes Jónsson. A climbing image nudged elastic band method for finding saddle points and minimum energy paths. *The Journal of Chemical Physics*, 113(22):9901–9904, 2000.

[5] Daniel Sheppard, Rye Terrell, and Graeme Henkelman. Optimization methods for finding minimum energy paths. *The Journal of Chemical Physics*, 128(13), 2008.

[6] Graeme Henkelman and Hannes Jónsson. Improved tangent estimate in the nudged elastic band method for finding minimum energy paths and saddle points. *The Journal of Chemical Physics*, 113(22):9978–9985, 2000.

[7] Hao Zhou, Matthew Grasinger, Philip Buskohl, and Kaushik Bhattacharya. Low energy fold paths in multistable origami structures. *International Journal of Solids and Structures*, 265:112125, 2023.

[8] XN Liu, GK Hu, CT Sun, and GL Huang. Wave propagation characterization and design of two-dimensional elastic chiral metacomposite. *Journal of Sound and Vibration*, 330(11):2536–2553, 2011.
