# Supplementary material for: Zero modes activation to reconcile floppiness, rigidity, and multistability into an all-in-one class of reprogrammable metamaterials
Source: Nat Commun. 2024 Apr 10;15:3087. doi: 10.1038/s41467-024-47180-0 (PMC11006655; doi:10.1038/s41467-024-47180-0)
Supplement: Supplementary file 3 — Description of Additional Supplementary Files [file 41467_2024_47180_MOESM3_ESM.pdf]

Description of Additional Supplementary Files

File Name: Supplementary Movie 1
Description: Activation and deactivation of the metahinge

File Name: Supplementary Movie 2
Description: Isochoric reconfiguration of the Kagome-type metamaterial capable of substantial stiffness reprogramming

File Name: Supplementary Movie 3
Description: Harnessing biaxial zero modes in a selectively activated Kagome metamaterial to transmit mechanical signals

File Name: Supplementary Movie 4
Description: Achieving mechanical logic operations through the biaxial zero mode

File Name: Supplementary Movie 5
Description: Multistable transition of a selectively activated Kagome metamaterial

File Name: Supplementary Movie 6
Description: Reprogramming the buckling mode in a rotation-square metamaterial via selective activation of metahinges

File Name: Supplementary Movie 7
Description: Non-isochoric reconfiguration of the metamaterial bearing metahinges with a coordination number of three

File Name: Supplementary Movie 8
Description: Non-isochoric reconfiguration of the metamaterial bearing metahinges with a coordination number of four
